# HiCeekR: A Novel Shiny App for Hi-C Data Analysis

**DOI:** 10.3389/fgene.2019.01079

**Published:** 2019-11-04

**Authors:** Lucio Di Filippo, Dario Righelli, Miriam Gagliardi, Maria Rosaria Matarazzo, Claudia Angelini

**Affiliations:** ^1^Telethon Institute of Genetics and Medicine (TIGEM), Pozzuoli, Italy; ^2^Istituto per le Applicazioni del Calcolo “Mauro Picone,” Consiglio Nazionale delle Ricerche, Napoli, Italy; ^3^Max Planck Institute for Psychiatry, Munich, Germany; ^4^Institute of Genetics and Biophysics “A. Buzzati A. Traverso,” Consiglio Nazionale delle Ricerche, Napoli, Italy

**Keywords:** Hi-C, user-friendly interface, long-range interactions, genome organization, topologically associating domains

## Abstract

The High-throughput Chromosome Conformation Capture (Hi-C) technique combines the power of the Next Generation Sequencing technologies with chromosome conformation capture approach to study the 3D chromatin organization at the genome-wide scale. Although such a technique is quite recent, many tools are already available for pre-processing and analyzing Hi-C data, allowing to identify chromatin loops, topological associating domains and A/B compartments. However, only a few of them provide an exhaustive analysis pipeline or allow to easily integrate and visualize other omic layers. Moreover, most of the available tools are designed for expert users, who have great confidence with command-line applications. In this paper, we present HiCeekR (https://github.com/lucidif/HiCeekR), a novel R Graphical User Interface (GUI) that allows researchers to easily perform a complete Hi-C data analysis. With the aid of the Shiny libraries, it integrates several R/Bioconductor packages for Hi-C data analysis and visualization, guiding the user during the entire process. Here, we describe its architecture and functionalities, then illustrate its capabilities using a publicly available dataset.

## Introduction

The DNA is organized in a three-dimensional (3D) structure inside the cell nucleus, where chromosomes occupy distinct regions called chromosome territories. Within chromosome territories, the chromatin forms Topological Associated Domains (TADs) characterized by a high frequency of intra-domain loci interactions. Inside the TADs, chromatin loops contain active genes and are physically separated from repressed domains. Investigating the 3D organization of chromatin is important to better understand the higher-order regulation of gene expression and, more in general, the genome functionality.

In the last twenty years, the advent of modern high-throughput technologies has allowed investigating chromatin structure and its hierarchical organization from an individual gene location to the global genome-wide perspective, using either method based on microscopy, such as fluorescent *in situ* hybridization (Solovei et al., 2002), and/or those based on chromosome conformation capture and their evolution. In particular, the original Chromosome Conformation Capture (3C) technique (Dekker, 2002), defined as *One-By-One* approach, enabled to study the 3D chromatin interaction between one region of interest and another single locus that is distant in the linear genome. Over the years, it was improved to expand the number of genomic regions studied in each experiment. Therefore, the Circular Chromosome Conformation Capture (4C) (Zhao et al., 2006) technique was proposed to investigate one locus of interest against all others (i.e. *One-By-All* approach), and later, the Chromosome Conformation Capture Carcon Copy (5C) (Dostie et al., 2006) allowed studying the interactions between multiple sequences (i.e. *Many-By-Many* approach). More recently, by combining proximity-based ligation with massively parallel sequencing, the High-throughput Chromosome Conformation Capture (Hi-C) (Belton et al., 2012; Dekker et al., 2013) allows to simultaneously investigate all genome interactions, therefore providing the *All-By-All* approach. Thanks to Hi-C experiments, it is now possible to study long-range interactions, i.e. physical interactions between chromosomal regions linearly distant that occupy the same spatial location in 3D chromatin conformation, identify chromatin hierarchical structures, and provide high-resolution 3D images of the chromatin architecture and its changes associated to diseases or treatments. However, to comprehensively explore the chromatin structure and its state, the integration of Hi-C results with the global epigenetic landscape is required. Due to the huge amount of data produced during Hi-C experiments, complex work-flows, and sophisticated computational algorithms are necessary to extract information and support the researchers in the interpretation of their computational results. Furthermore, these workflows need to be adapted, in terms of resolution and algorithms, to the specific structures of interest, see Nicoletti et al. (2018); Pal et al. (2019) for general views.

The first step of the data analysis consists of the alignment of the raw reads on a reference genome. However, due to the presence of DNA fragments originated from two distinct genomic loci, that are combined during ligation, the two mates are usually aligned independently and the mapper often requires to incorporate an iterative procedure to better identify the ligation junction. Tools such as HiCUP (Wingett et al., 2015) or the iterative approach described in Imakaev et al. (2012) can be used, instead of classical short-read mappers. The alignment step produces Binary Alignment Map (BAM) files containing the genomic coordinates of each aligned read on the chosen genome. Such files need to be filtered to remove spurious sequences, PCR duplicates, digestion or ligation artifacts, low-quality sequences, and any other sources of technical noise from the sequences of interest.

The analysis is then carried on the retained high-quality sequences. The reference genome is divided into small regions (called bins), that are used to evaluate a square symmetric matrix (known as raw contact matrix) by counting the number of paired-end reads inside each pair of bins. Such a step is often referred to as binning and the contact matrix measures the strength of the interaction between two bins (i.e. the rows and the columns of the contact matrix). The bin width defines the resolution of analysis and, as a consequence, the computational time and the resources required to perform the analysis. The choice of the resolution depends on the organism under investigation, the sequencing depth, the size of the restriction fragment, as well as the available computational resources.

Subsequently, the contact matrix has to be normalized to mitigate bias effects typically present in this type of analysis. Normalization is a crucial step that can have a strong effect on the results (Ay and Noble, 2015). Some normalization algorithms were proposed in Yaffe and Tanay (2011); Hu et al. (2012); Imakaev et al. (2012); Knight and Ruiz (2013). The normalized contact matrices are useful for visualization and are used for further downstream analysis.

The post-processing or downstream analysis defines a wide series of computational procedures aimed at identifying and extracting hierarchical chromatin structures of interest. For example, it is possible to partition the genome in compartments, usually denoted as A and B compartments. Such domains are usually located along the same chromosome and display strong interactions within the same domain and negligible interactions with the other domains. It has been shown that such compartments are connected to active and inactive chromatin states, respectively, and can be related to regions of (gene-dense) euchromatin and regions of (gene-poor) heterochromatin. Compartments are usually identified at a resolution of 100 Kbp or higher. Moreover, by looking at the block-wise structure of the contact matrix, contiguous regions of high self-interactions clearly separated from adjacent regions can be identified. Such regions are usually referred to as tad and the separation boundaries determine their coordinates. tad are usually identified with a resolution of 50 Kbp or higher. Several methods have been proposed for identifying tad boundaries, see Zufferey et al. (2018). With higher-resolution analysis, it is possible to identify specific point-to-point interactions usually referred to as loops. Such interactions can be either *cis*-interactions or *trans*-interactions and appear as spike signals in the contact map. Loops are usually identified with a resolution of 10 Kbp.

Finally, it is also helpful to integrate hic data with other experimental genome-wide datasets [i.e. Chromatin Immunoprecipitation Sequencing (ChIP-Seq) or RNA sequencing (RNA-Seq)] or with other information from an external database to support the researcher in interpreting experimental data, provide evidence of specific regulatory mechanisms and/or insight for novel research hypotheses.

In the last few years, several computational approaches have been proposed to either to perform one or few of the above-mentioned steps or to combine them in more general pipelines. From one hand, the interesting comparative study made in Forcato et al. (2017) provided a clear and detailed description of the advantages and drawbacks of individual methods/algorithms. Indeed, after bench-marking several procedures using different quality indexes, Forcato et al. (2017) showed that several methods reported good performance on some specific steps, although no methods outperformed the others. On the other hand, despite the great effort in the development of tools specifically designed for the analysis of Hi-C, they rarely include all the required functionalities for complete analysis in a single platform. Han and Wei (2017) and Calandrelli et al. (2018) provided a recent list of existing general-purpose tools. In general, most of the available tools are designed for expert users with great confidence about command-line applications. As a consequence, they are not supporting user-friendly data explorations that can lead experimental biologists to easily interpret their results, confirm, or make novel scientific hypotheses. These motivations led us to the development of HiCeekR, a novel computational tool that allows performing most of the above-mentioned steps, through an easy user-friendly graphical interface, combining different algorithms for the analysis of Hi-C data. Moreover, HiCeekR has been designed for guiding the users during the entire analysis process and to provide interactive plots that might help researchers with limited experience in command-line applications, to explore and visualize data and results using a simple *point-and-click* approach.

## Materials and Methods

In this section, we first describe HiCeekR workflow, then we provide technical details about its implementation and the structure of the Graphical User Interface (GUI). Finally, we illustrate how HiCeekR stores input/output data and results, and describe the internal modular architecture.

### HiCeekR Workflow

HiCeekR is a novel Shiny based R package (https://github.com/lucidif/HiCeekR) for Hi-C data analysis. Thanks to its GUI, HiCeekR friendly guides the user during the entire analysis process, allowing him/her to perform a complete data analysis pipeline and to integrate Hi-C data with other omic datasets. Moreover, HiCeekR produces several interactive graphics that allow exploring the results by the usage of the mouse pointer.

**Figure 1:**
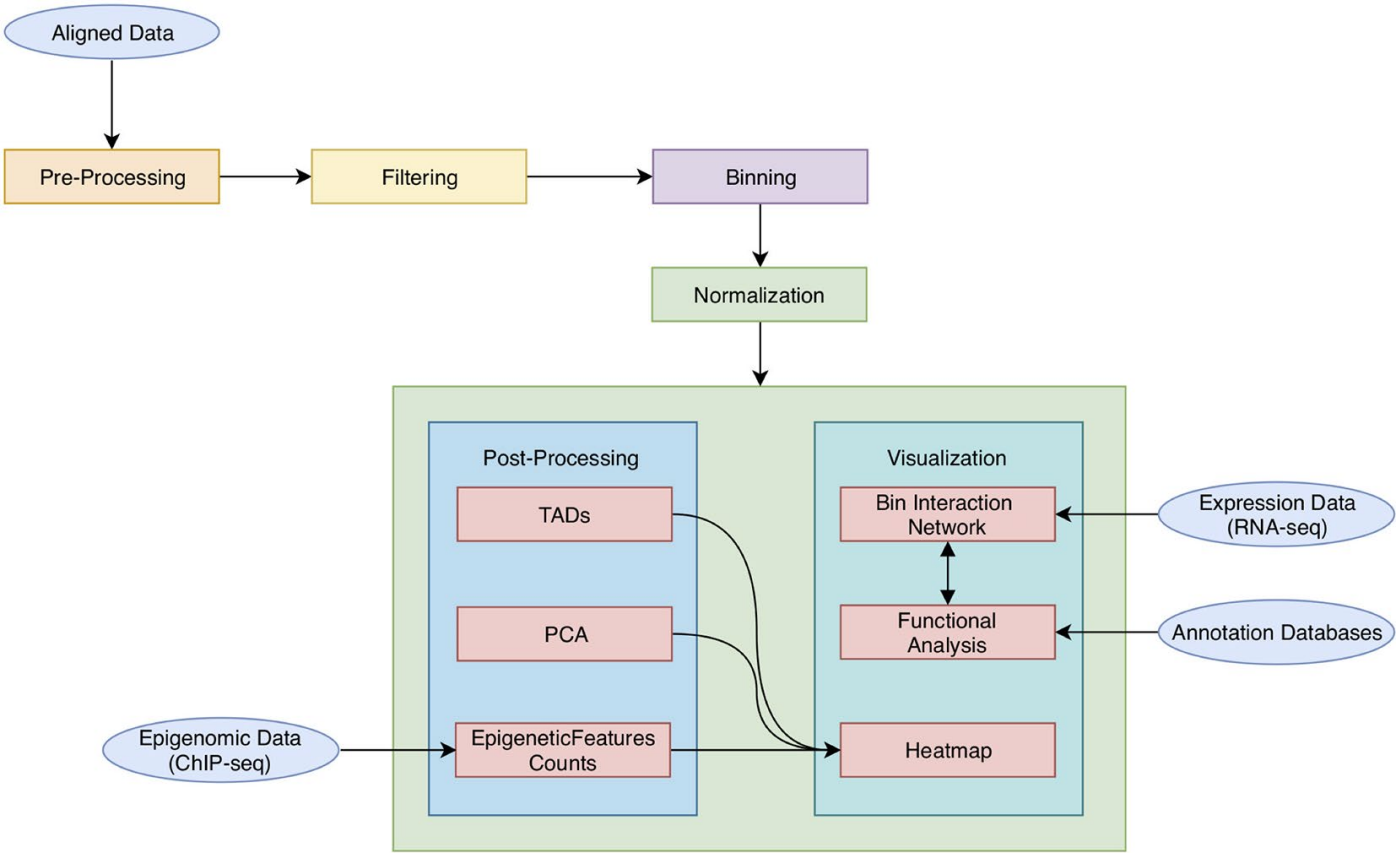
A schematic representation of HiCeekR pipeline. Starting from aligned data, HiCeekR enables to pre-process and filter them to compute (and normalize) the contact matrix. Afterward, it performs several downstream analysis steps in order to detect genome compartments, TADs. Moreover, it also allows the integration of additional epigenetic and transcriptional whole genome datasets, as well as other genome-wide tracks. Finally, it presents the results in interactive graphical forms.

As shown in **Figure 1**, HiCeekR analysis starts from already aligned sequence files (in BAM format) obtained from Hi-C experiments, it proceeds through a series of steps from pre-processing and filtering, to the evaluation and normalization of the contact matrices. Once the contact matrices are available, the user can perform the downstream analysis. In particular, HiCeekR allows the identification of genome compartments and tad, the integration of Hi-C data with other omic datasets, such as ChIP-Seq and/or RNA-Seq, the functional analysis, and the visualization of the interaction network. Overall, HiCeekR supports the user in elucidating the functional interplay between chromatin structure and gene regulation by combining and making friendly available a wide bunch of computational and statistical methods.

Through HiCeekR, each step/function can be executed sequentially in a step-by-step analysis (**Figure 1**). After each step, the user can visualize intermediate results, such as summary statistics or graphical representations. However, each step or function can be re-executed by modifying the parameter settings, obtaining consequently updated results. Intermediate and final results (as text files or figures) are stored in pre-organized data structures (see *Data Format and Data Organization*) that can be easily retrieved for future investigations through the HiCeekR GUI.

#### Pre-Processing

The pre-processing consists of a series of fundamental operations required for the proper execution of HiCeekR. Such operations allow HiCeekR to easily access the information in the subsequent steps and are aimed to reduce the overall execution time. In HiCeekR, the pre-processing is jointly performed with the creation of a new project (see *Getting Started for the Analysis*), when the user selects the experimental Hi-C files (in BAM format) to work on and the reference genome (in *FASTA* format). At this stage, it is also required to provide the restriction enzyme cutting site and an overhang parameter (in base pairs) that are necessary to split the genome in restriction fragments. The overhang parameter defines the number of base pairs overlapping the restriction enzyme cutting site. Given such information, the restriction fragments are indexed. The coordinates of each detected restriction enzyme cutting site are stored in an index-file (HDF5 file) and associated with one or more mapped read allowing to speed up further computations. The HDF5 file format (https://www.hdfgroup.org/solutions/hdf5/) is chosen for speeding-up heterogeneous data storage and processing, and it is not usually meant to be inspected by a standard user. Note that at this stage, low-quality reads are automatically removed.

**Figure 2:**
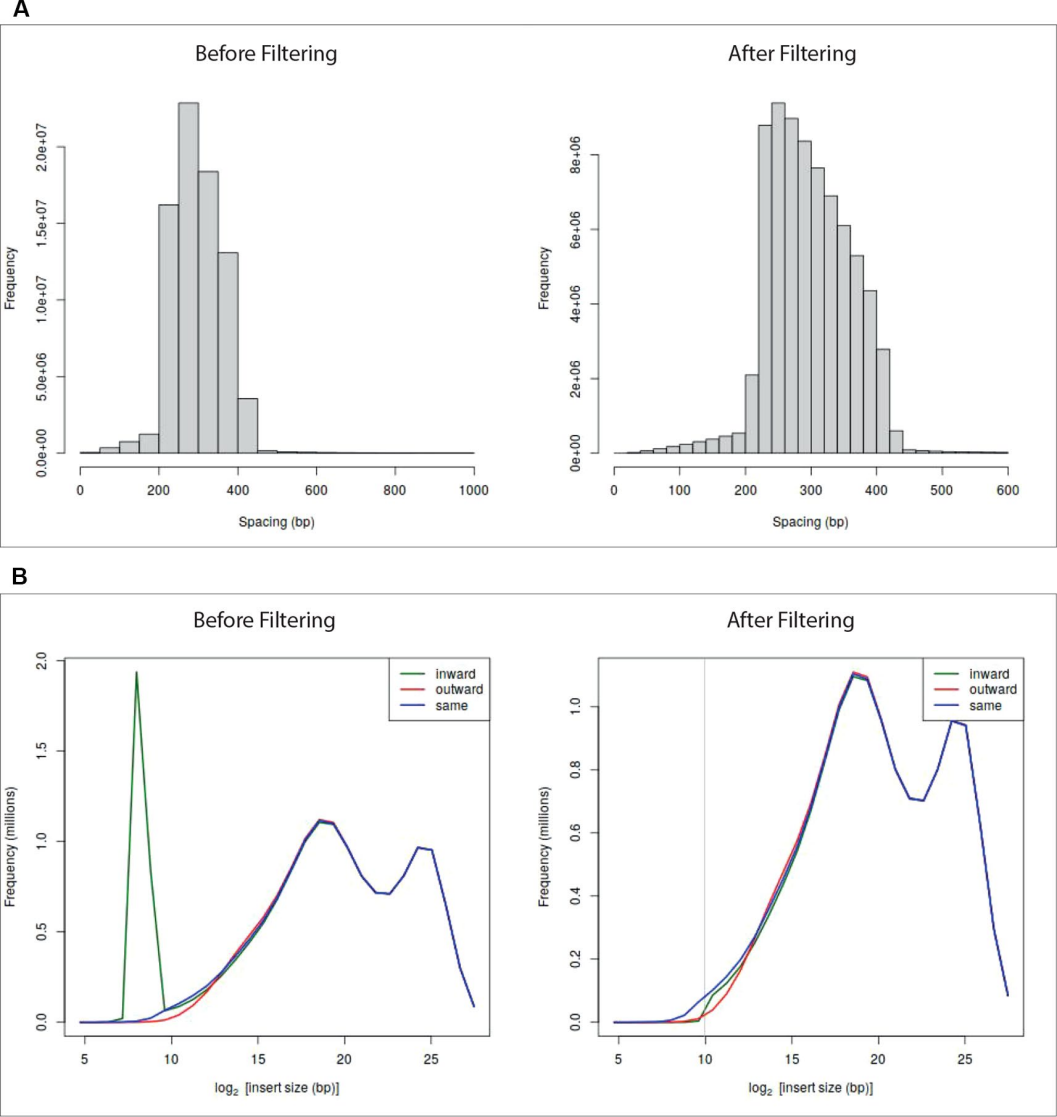
Diagnostic plots and effect of the filtering on sample GSM1608509 (see *A Case Study*). Panel **(A)** shows read length distribution before and after filtering. The plot before filtering indicates that long fragments are present, the corresponding plot after filtering shows that fragments larger than 600 bp were removed. Panel **(B)** shows the read-orientation plot before and after filtering. The plot before filtering suggests possible dangling-end events (green line spike) located at about 2^8^  =  256 bp, the corresponding plot after filtering shows that such inward-oriented pair of reads were removed.

At the end of the pre-processing, HiCeekR produces a summary of the statistics for the indexed reads and two diagnostic plots (see **Figures 2A, B**—before filtering) useful to detect artifacts that will be removed during the filtering step. The first plot represents a distribution of the insert lengths over the entire genome, the second shows the distribution of the inward-outward insertion lengths (see *Filtering* for further details).

Additionally, during the pre-processing, HiCeekR defines the resolution of the entire analysis by the selection of the bin size (default is 6) base pairs), that is used afterward during the binning step.

#### Filtering

The filtering step is aimed to remove well-recognized artifacts that are produced during library preparation, such as PCR-artifacts, self-circle, and dangling-end fragments (Belton et al., 2012; Ay and Noble, 2015; Lajoie et al., 2015).

In particular, HiCeekR automatically removes PCR duplicates, when previously marked in the BAM files. Marking duplicates can be easily carried out using standard tools.

The identification of self-circle and dangling-end fragments is obtained from the association between read-pairs and restriction fragments that can lead to a two case scenario: the read-pair is associated to different restriction fragments or the same restriction fragment. The former case constitutes the set of valid reads, while the latter occurs when un-ligated dangling-end or circularized self-circle fragments are present into the library preparation. Self-circle (outward strand orientation) and dangling-end (inward strand orientation) fragments can be discriminated each other by looking at the strand orientation of the paired-reads that fall in the same restriction fragment. Since such read-pairs are considered uninformative, they are removed during the filtering step.

HiCeekR removes self-circle and dangling-end fragments by setting a minimum distance for inward pair reads and outward pair reads (*min-inward* and *min-outward* values). It calculates the distance of each associated read from the nearest restriction enzyme site and then estimates the length of the sequencing fragment. Very long fragments, that could be associated with unwanted ligation products, can also be removed by setting a suitable threshold through the *max-frag-length* parameter. By inspecting the diagnostic plot in **Figures 2B**—before filtering), the user can select the *min-inward* and *min-outward* values to remove self-circle and dangling-end products (Lun and Smyth, 2015).

After the filtering process, HiCeekR updates the diagnostic plots (**Figure 2**—after filtering). Results are stored in an HDF5 format.

#### Binning

The binning step is aimed to perform all those operations required to evaluate the raw contact matrix (Ay and Noble, 2015). To this purpose, the reference genome is divided into *n* *_b_* bins of approximately non-overlapping and fixed-width *w* *_b_* (fixed-size bin). Indeed, the exact bin subdivision depends on the locations of the restriction enzyme cutting sites, and few bases of overlap might be allowed between consecutive bins. We recall that the bin size *w* *_b_* determines the resolution of the analysis (also the resources and the required running time). It is important to select *w* *_b_* to guarantee good statistical power at an affordable computational cost. Unfortunately, there are no precise guidelines for the selection of *w* *_b_*, since its choice depends on the sequencing depth and the type of chromatin structure of interest. For these reasons, HiCeekR allows the user to perform the computational analysis at different resolutions, suggesting to first use a low resolution to obtain a general view of the chromatin organization and then repeating and refining the analysis by increasing the resolution while focusing on specific genomic locations of interest (for example, a specific chromosome, or a specific sub-region or two sub-regions located on different chromosomes).

After the bins indexing, HiCeekR assigns the previously filtered-in reads to the genome bins where they better map. Then, it produces the raw contact matrix, a symmetric square matrix $M\in R^{n_{b}\times n_{b}}$, by counting the number of reads *M* *_i,j_* that fall within the bins *i* and *j*, respectively. To facilitate data exploration, the indexed bins are automatically converted into genomic coordinates. By exploring the raw contact matrix, it is common to observe bins with very large/small values that appear as “outliers” and might due to noise such as low mappability or the presence of many repeated sequences. To reduce this problem, it could be useful to remove “outliers” bins by using a bin-level filtering strategy, as suggested by Lajoie et al. (2015). However, such “outliers” bins can be detected in different ways (Lajoie et al., 2015). The current version of HiCeekR does not implement any bin-level filtering, although we plan to integrate such functionality in future releases.

At the end of binning, HiCeekR stores the bins genomic coordinates as a BED file format and the entire count matrix as a Tab Separated Valuer (TSV) file.

#### Normalization

The normalization step is aimed to remove technical bias from the raw contact matrix that could lead to false positive/negative findings. The output of such step is a normalized contact matrix, a symmetric square matrix $\hat{M}\in R^{n_{b}\times n_{b}}$ of real values, that constitutes one of the main results of the computational data analysis. The current release of HiCeekR implements two different strategies for normalizing the contact matrix: the iterative correction and eigenvector decomposition (ICE) (Imakaev et al., 2012), and the WavSiS (Shavit and Lio’, 2014).

ICE is a well-known correction method based on the assumption that the bias in the interaction between two loci can be factorized as the product of the individual biases, affecting each of the two interacting loci (Imakaev et al., 2012). By using such matrix factorization approach, ICE method applies an iterative decomposition algorithm based on the maximum likelihood to convert the raw contact matrix into a normalized one of relative contact probabilities, guaranteeing equal visibility for each region. In particular, the ICE method gives the possibility to Winsorize the matrix to mitigate the effect of the impact of high-abundance bin pairs by using the *Winsor.high* parameter, in combination with the *ignore.low* parameter to not ignore the low abundance bins.

WavSis removes noise by inspecting the variance distribution of the coverage across different physical scales, stabilizing the variance, and applying a wavelet denoising strategy. In particular, the raw contact matrix *M* (whose entries *M* *_i,j_* are assumed to follow a Poisson distribution) is regarded as a series of decomposed vector coefficients (whose number depends on the number of chromosomes), using the Haar-Fisz transform, which helps in stabilizing the variance. After that, a Gaussian wavelet shrinkage method is used to remove the noise from each set of coefficients and the normalized matrix is reconstructed by inverting the transform. This method is performed independently on each chromosome (selected through the *chromosome of interest* select-box). Additionally, it is possible to remove uncovered regions (detected during this normalization phase) with *NA* values, by using the *remove uncovered* checkbox.

At the end of this process, HiCeekR generates a new tsv file with the normalized count matrix.

#### Post-Processing

HiCeekR post-processing or downstream analysis supports the user in extracting chromatin structures from the raw or normalized contact matrix and interpreting the results in multiple ways: the detection of A/B-compartments and TADs, the integration with other omic-layers, and the functional interpretation, respectively. These functionalities are available to the user through the modules PCA, directionality index, TopDomTADs, HiCsegTADs, EpigeneticFeatures, and bed2track (in the Post-processing panel), Heatmap, and Network (in the Visualization panel).

HiCeekR detects A/B compartments thanks to the PCA module that performs the principal component analysis (PCA). Large-scale interaction patterns can be identified from the normalized contact matrix by computing the preferential interacting regions (the so-called, compartment A and compartment B). The compartments can be identified by looking at the PCA eigenvector with opposite signs (Lieberman-Aiden et al., 2009; Lajoie et al., 2015). This step requires to select the normalized contact matrix and outputs the PCA eigenvectors (stored as PCA eigenvector matrix) that can be used either to define compartments and for visualization purposes (**Figure 6**). Usually, the first one or two PCA eigenvectors are sufficient to identify the compartments.

Current version of HiCeekR highlights the TADs using three approaches: i) directionality index, ii) TopDom, and iii) HiCseg.

The directionality index module computes the directionality index *d* *_i_*, as introduced by Dixon et al. (2012). *d* *_i_* is defined as

$$\begin{array}{cc} d_{i}=\left(\frac{b_{i}-a_{i}}{\left|a_{i}-b_{i}\right|}\right)\left(\frac{{\left(a_{i}-e_{i}\right)}^{2}}{e_{i}}+\frac{{\left(b_{i}-e_{i}\right)}^{2}}{e_{i}}\right), & i=1,.\ldots ,n_{b} \end{array}$$

where *a* *_i_* and *b* *_i_* denote the number of mapped reads in the upstream and in the downstream of bin *w* *_i_*, respectively, and $e_{i}=\frac{a_{i}+b_{i}}{2}$. The directionality index *d* *_i_* generates a segmentation of the genome, and the TADs are defined as the regions between two sharp changes of directions in such indexes.

The TopDomTADs module implements TopDom algorithm, as proposed in Shin et al. (2015). In particular, it defines a segmentation of the genome based on a three steps procedure: it evaluates the contact frequency signal as the average contact frequency of each bin with its upstream or downstream regions, then selects potential TADs boundaries as the local minima of the contact frequency signal, finally it filters out potential false positive by using Wilcox Rank Sum test under the assumption that the expected contact frequencies of regions within a TADs should be higher than those of a bin in the TADs and a bin outside the TADs, and of those bins outside the TADs. The number of bins to be included in upstream or downstream regions can be controlled by the user with the parameter *Window Size*, which constitute the only tuning parameter of TopDom algorithm.

The HiCsegTADs module implements HiCseg algorithm, as proposed in Lévy-Leduc et al. (2014). In particular, it defines a partition on the contact matrix (either the raw matrix *M* or the normalized contact matrix $\hat{M}$) with a block structure depending on the unknown TADs boundaries. The parameters of the distributions are estimated by a maximum likelihood approach assuming that the observed contact values, *M* *_i,j_* or ${\hat{M}}_{i,j}$, within the same TADs share the same distribution parameters. Maximum likelihood estimates are obtained using a dynamic programming algorithm. In this context, Gaussian distributions have to use for modeling normalized contact matrix $\hat{M}$, whereas Poisson or Negative binomial distributions for raw contact matrix *M*. The user can also choose the maximum number of TADs with the parameter *Kmax* and the structure (i.e. block-diagonal or extended-black diagonal) of the matrix segmentation.

At the end of the TADs processing, HiCeekR automatically generates output files as directionality index track (as a coverage file), and the detected TADs boundaries (in standard BED format). Note that for all modules, the identification of compartments and TADs is performed independently for each chromosome.

As already mentioned, one of the advantages of HiCeekR is given by the possibility to integrate and visualize Hi-C data together with other omic data. To this purpose, in the EpigeneticFeatures module, it is possible to upload one or more aligned BAM files from ChIP-Seq experiments. Then, HiCeekR computes the normalized coverage at the same bin-width resolution chosen for the current Hi-C analysis. Mimicking classical ChIP-Seq coverage, the normalized coverage can be computed either as the number of reads within the bin per million of mapped reads (RPM) or the ratio of the number of reads within the bin in the ChIP-Seq sample over those in the input DNA sample. Additionally, with the bed2track module, it is also possible to process any other genome-wide track in BED format. Such track will be converted by HiCeekR in bin coordinates (i.e. the bin coordinates will be included in the converted track when they intersected the user supplied BED track) to be visualized.

Note that, thanks to the Heatmap module, the user can visualize the normalized contact matrix, the PCA loadings, and/or the directionality index *d* *_i_*, and/or any bed track (such as those provided as output by TopDomTADs or HiCsegTADs, or converted from user supplied tracks using bed2track), then can add one or more ChIP-Seq coverage tracks to have a more detailed overview of the chromatin state (**Figure 6**).

Finally, in the Network analysis, HiCeekR automatically retrieves the list of genes located within a specific compartment, TADs, or regions of interest. The annotation is obtained overlapping the bins coordinates of the region of interest with the genomic coordinates of the genes (as provided in an annotation file). To this purpose, note that a given bin might be associated with several genes (if the bin overlaps the gene body of more genes), or a given gene might be associated with multiple bins if its gene body is larger than the bin resolution or it overlaps any bin boundary. There are bins not containing genes. The gene-bin association map depends on the annotation and the resolution of the analysis. HiCeekR provides three interactive tables Interaction, Genes, and Enrich. Interaction is a table that contains, for each pair of interacting bins, the corresponding genomic coordinates, the interaction strength, the names of the genes therein contained (if any), and few other information. The gene symbols are hyperlinked to GeneCards (https://www.genecards.org) to facilitate the data interpretation. In the Enrich table, the results of the functional analysis on the identified genes carried out using gProfiler are shown (Raudvere et al., 2019). Identified enriched GO terms, or KEGG and Reactome Pathways are reported together with enriched regulatory motifs/transcription factors (from TRANSFAC), tissue specificity (from human protein Atlas database), Human-specific phenotypes (from Human Phenotype Ontology Database), protein complexes 267 (from CORUM) and results from other interrogated databases. Genes is a table that, among several other information, allows to visualize the gene expression values of the identified genes (only if the user uploaded a gene expression dataset either from RNA-Seq or microarray experiments) that can help in better discriminating chromatin states.

#### Visualization

**Figure 3:**
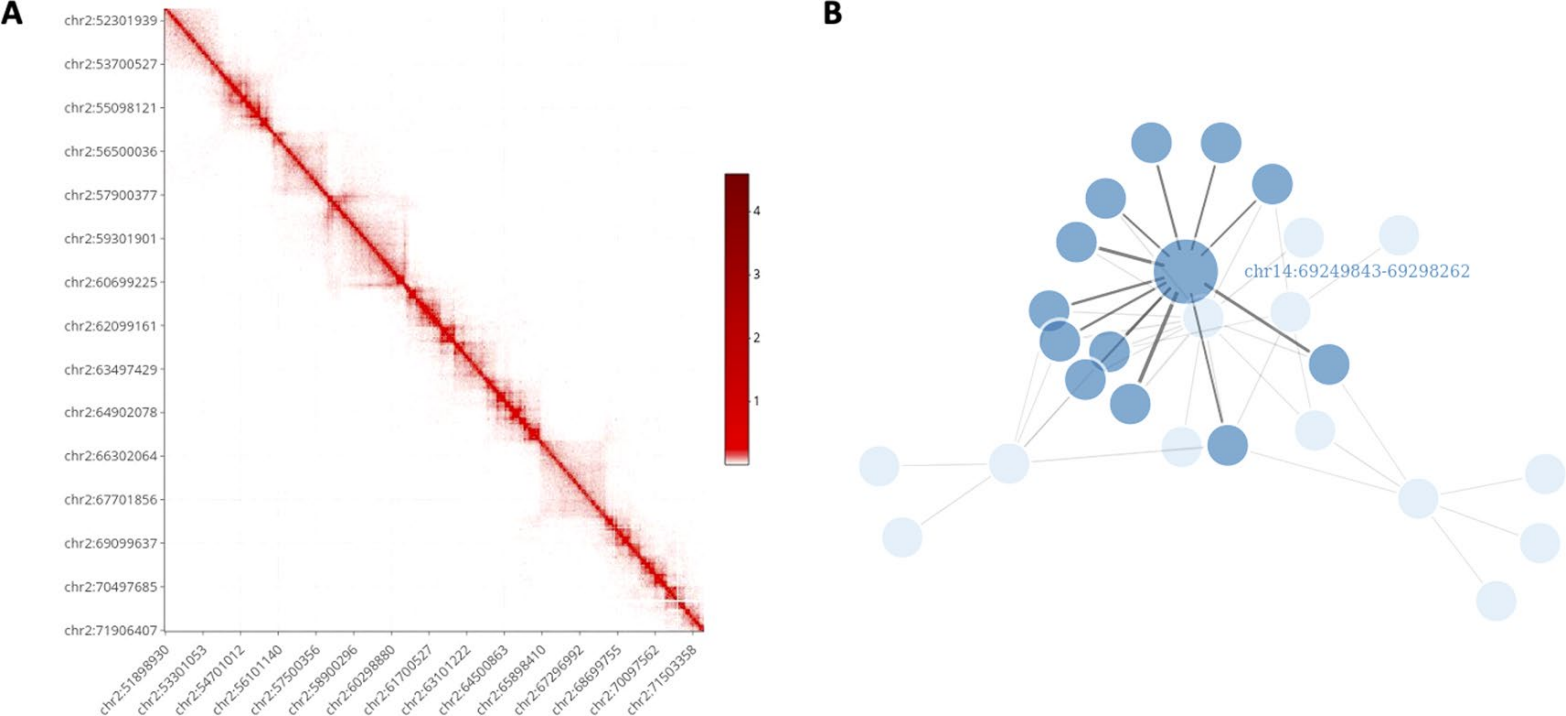
HiCeekR graphical output. **(A)** Heatmap representation on the contact matrix. **(B)** Network representation of selected contacts.

It is well known that the visualization of information in a graphical form constitutes one of the most important data exploration tools. However, visualizing Hi-C data can be challenging due to the high-dimensionality of the files and the dimension of the genome. Nowadays, several visualization tools are available, see Yardimci and Noble (2017) for a general review. Nevertheless, HiCeekR provides functions to visualize the obtained results without requiring additional software. Moreover, most of the HiCeekR plots are interactive. In particular, the user can select two main representations: *Heatmap* and *Network* (**Figure 3**).

Using the *Heatmap visualization* the user can explore the raw and the normalized contact matrix using the classic heatmap graphical representation where low and high contact values are depicted using different color intensities. He/she can select a specific chromosome or a pair of chromosomes or, otherwise, a region of interest within each of them. Moreover, it is possible to zoom in/out or to move to another region of interest. Additionally, in the *Heatmap visualization*, the user can add several other genome-wide *tracks* that allow to simultaneously visualize multiple information, such as the loadings of the PCA, the directionality index *d* *_i_*, any BED format track (i.e. generated by the TADs modules or converted by bed2track module) as well as other omic profiles, such as ChIP-Seq profiles, on the same genome-wide scale, as shown in **Figure 6**.

Using the *Networks visualization* the users can visualize the interactions of a set of bins of interest against all other bins in network form, where the vertices represent the bins and the edges represent the detected interactions. Moreover, the link width is proportional to the strength of the interaction. Additionally, by using user-defined cut-offs, it is to possible to filter-out negligible interactions.

### Implementation

HiCeekR is an R-Shiny web GUI which combines several R/Bioconductor packages widely used for Hi-C data analysis and visualization functionalities. In particular, the filtering and the binning steps are implemented using diffHic package (Lun and Smyth, 2015), one of the most used tools for this type of data. Matrix normalization is carried out using ChromeR package (Shavit and Lio’, 2014) for the WavSis method and diffHic for the ICE algorithm. The downstream analysis is based on HiTC for the PCA and for the directionality index modules (Servant et al., 2012), TopDom for the TopDomTADs module, HiCseg for the HiCsegTADs module, gProfileR for functional enrichment, and other customized R functions. The graphical output is produced using the ggplot2, plotly, heatmaply, networkD3, and corrplot packages.

Finally, from the architectural point of view, HiCeekR is open-source, easily expandable with additional functionalities (thanks to the modular structure) and it also allows to integrate third-party functions, as discussed in *Shiny, Modules, and Other Technical Considerations*.

#### Graphical User Interface

**Figure 4:**
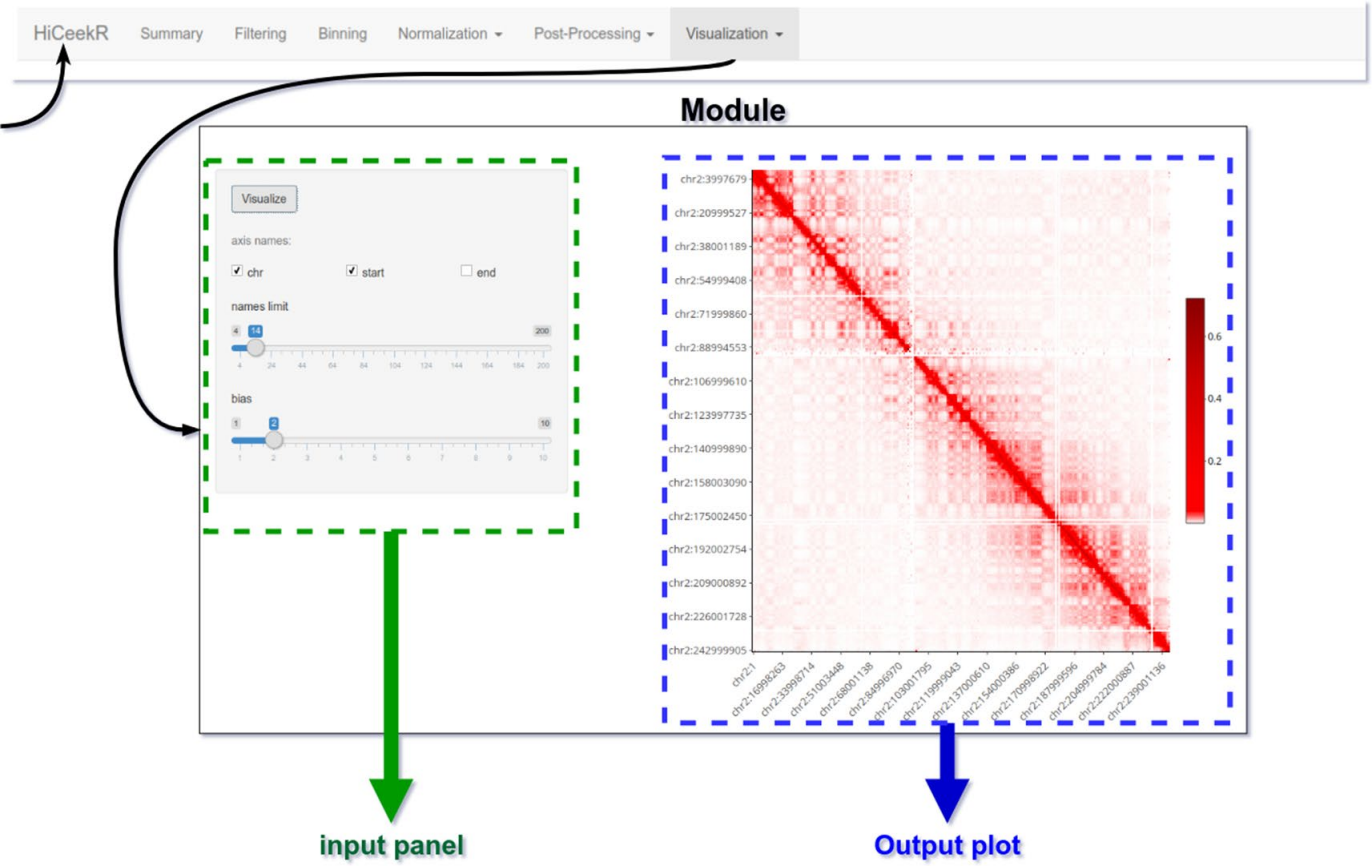
HiCeekR graphical interface. The upper part of the interface is the navigation bar; on the left side the user can select the parameters of the function, on the right side results will be displayed in form of tables or plots.

The graphical interface has been designed for guiding the user during the entire analysis process. To this purpose, as shown in **Figure 4**, the upper part of the interface displays the navigation bar illustrating all the main analysis steps in sequential order (i.e. Pre-Processing, Binning, Normalization, Post-Processing, Visualization). Each analysis step panel contains one or more specific functions. By selecting one of them, HiCeekR renders the “Function panel” where input data files, function parameters and/or options (default values are suggested whenever possible) can be set before executing the function (the left side of the interface allows the user to choose all the parameters/options). The results are shown in the “Result panel,” that is displayed on the right side of the interface, as plots or tables are automatically saved in a pre-structured way. The graphical representations are interactive and allow exploring the results through point&click and dragging&dropping approach.

#### Getting Started for the Analysis

At the first HiCeekR execution, the user has to create a configuration file. A dedicated interface will guide him/her by browsing the *working folder*. This step is mandatory for further analyses. Then, each time HiCeekR is executed, the user can either create a new data project or continue/update an already existing project (by selecting the *load* option in the *Welcome* interface). When an experimental dataset is analyzed for the first time, the user will create a new project. HiCeekR will create the data structure, as described in *Data Format and Data Organization* and later results will be stored in a corresponding project name folder. After that, the data analysis can be initiated.

#### Data Format and Data Organization

**Figure 5:**
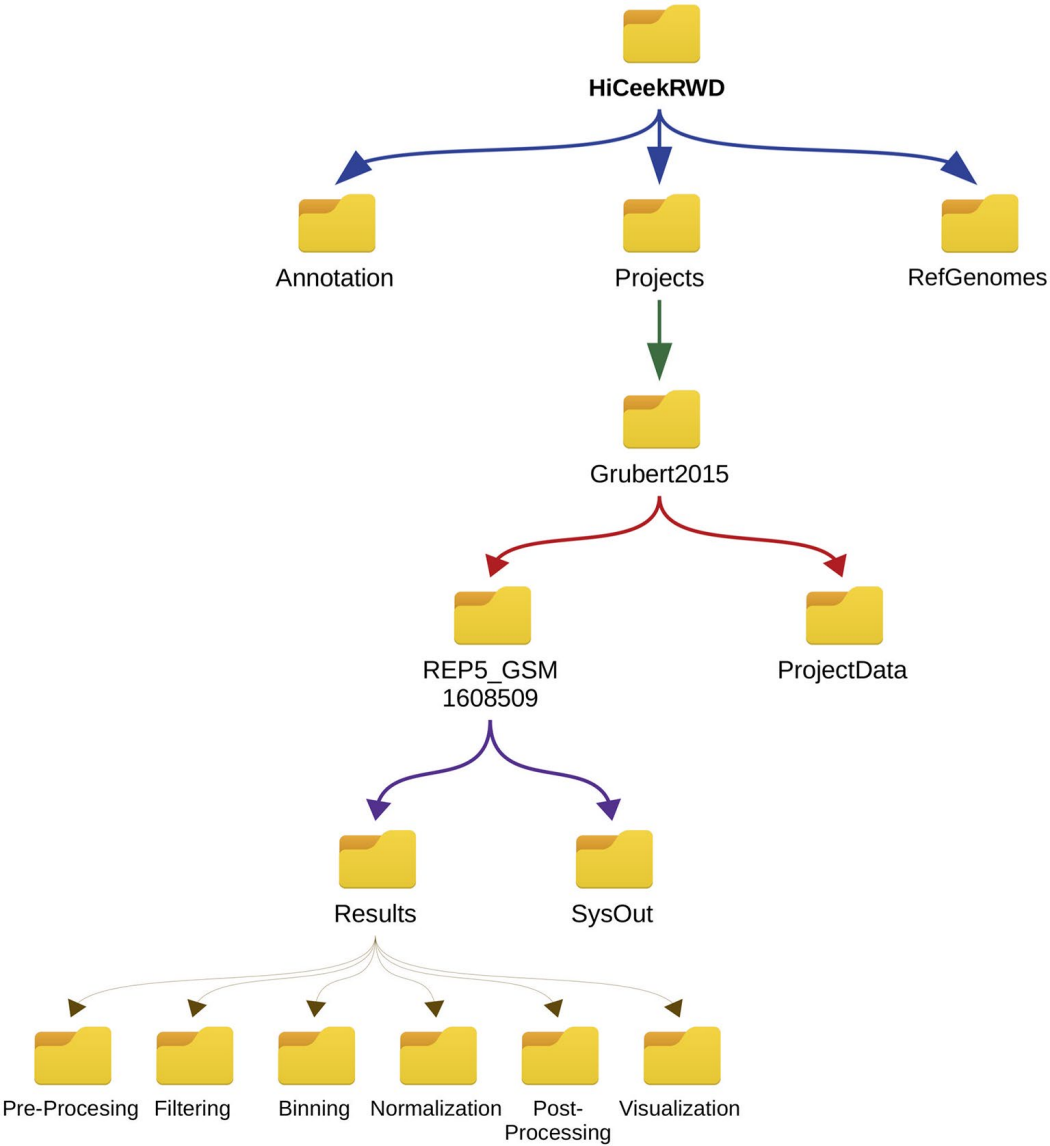
HiCeekR hierarchical data structure. All data are contained in the *HiCeekR* working directory folder and organized in projects. *HiCeekR* working directory folder is created the first time HiCeekR is executed, using the configuration file (see *Getting Started for the Analysis*). Genomes and Annotations can be shared across different projects and are stored in the *Genomes* and *Annotation* folders, respectively. All user projects are saved in the specific *Project_folder* contained in *HiCeekR_projects* main directory. Within the specific *Project_folder* it is possible to create sub-folders related to a specific sample, and/or analysis resolution. Each sub-folder contains *Results* and *SysOut* folders. Folder *Results* contains a sub-folder for each analysis step where intermediate and final results are saved. Folder *SysOut* contains internal logs file and it is not meant for standard users.

HiCeekR allows handling both user experimental data and other information such as the reference genome and annotations. Reference genomes are stored in the *Genomes* folder (in FASTA format), gene annotation in the *Annotation* folder [in Gene Transfer Format (GTF) format]. User experimental data mostly consist in Hi-C sequencing data (i.e. aligned BAM files) obtained from short-read alignment software. However, during the downstream analysis, HiCeekR can use other experimental data such as aligned sequences (i.e. BAM files) obtained from a ChIP-Seq analysis workflow or gene expression values (i.e. TSV file) obtained from RNA-Seq analysis pipeline. We stress that for these additional data the reference genome used during the alignment has to be consistent with the one used for aligning Hi-C data and the gene identifiers have to be consistent with those available in the annotation file. All user experimental data, that refers to the same project, are stored in the *Project data* folder contained in the specific *Project* folder, which has been created by HiCeekR during the pre-processing phase. All user project folders are saved in the *HiCeekR_projects* main directory. Within each *Project* folder, the results of a specific analysis are organized in the *Analysis* folder, different for each sequence file and resolution (i.e. the width *w* *_b_* chosen during the binning phase). During each analysis step, HiCeekR stores the results in files in corresponding sub-folders for the specific step. **Figure 5** shows the input/output data organization folder tree.

#### Shiny, Modules, and Other Technical Considerations

HiCeekR is implemented using R/Shiny library and modular structure. R/Shiny package easily allows developing advanced and practical interfaces in a web-based approach combined with the power of the R statistical instrument. Shiny apps were originally designed for small applications consisting of two main entities: the Shiny User Interface (SUI) that provides all the aesthetic components the user interacts with and the Shiny Server Side (SSS) that performs the required computations. Nevertheless, nowadays it is possible to implement complex applications by combining multiple modules.

A module is conceived as a shiny independent app, with its SSS and SUI. Each HiCeekR interface corresponds to a different module. Overall, the modular structure implemented in HiCeekR allows handling the complexity of the interface and better face the maintainability of the software, not only from a bug-fixing point of view but also when novel functionalities need to be added. Indeed, in this latter case, to add a novel module it will be necessary only incorporate the novel interface, which implements the required functionalities. Thanks to this choice, HiCeekR results in an easily expansible software.

### HiCeekR and Other Available Tools

As mentioned in the *Introduction*, there are relatively few tools that allow performing a comprehensive Hi-C data analysis [see, Calandrelli et al. (2018) and Han and Wei (2017)] for a short list of the most popular tools). Most of them are implemented either in Python, R, Perl, C++, or as a combination of different programming languages. Moreover, they often require several external dependencies to be installed. Out of them, GITAR Calandrelli et al. (2018) and HiCPro Servant et al. (2015) were implemented mostly in Python as command-line. They constitute two useful pipelines designed for expert users (i.e. they allow to perform a specific analysis step or a series of steps). However, they do not have a graphical interface supporting non-expert users. Similarly, HiC-bench Lazaris et al. (2017) provided a well-organized R/Python platform (with a large number of functionalities including those for parameter exploration), but has the same above-mentioned limits for the support of non-expert users. By contrast, HiCdat Schmid et al. (2015) and HiCexplorer Wolff et al. (2018) equipped their tool with a graphical interface. However, the interface of HiCdat is quite naive and limited to the pre-processing step (the higher-order analysis steps have to be performed as command-line). Vice versa, the interface of HiCexplorer is Galaxy based. Hence, it meets the needs of non-expert users as HiCeekR. However, HiCexplorer lacks interactivity in the graphical visualization. Moreover, its local installation is computational demanding. Compared to the above-mentioned alternatives, HiCeekR is completely R based, easy to install and presents a modular graphical interface designed for supporting non-expert users with several functions for interactive visualization of the results.

## Results

### A Case Study

**Figure 6:**
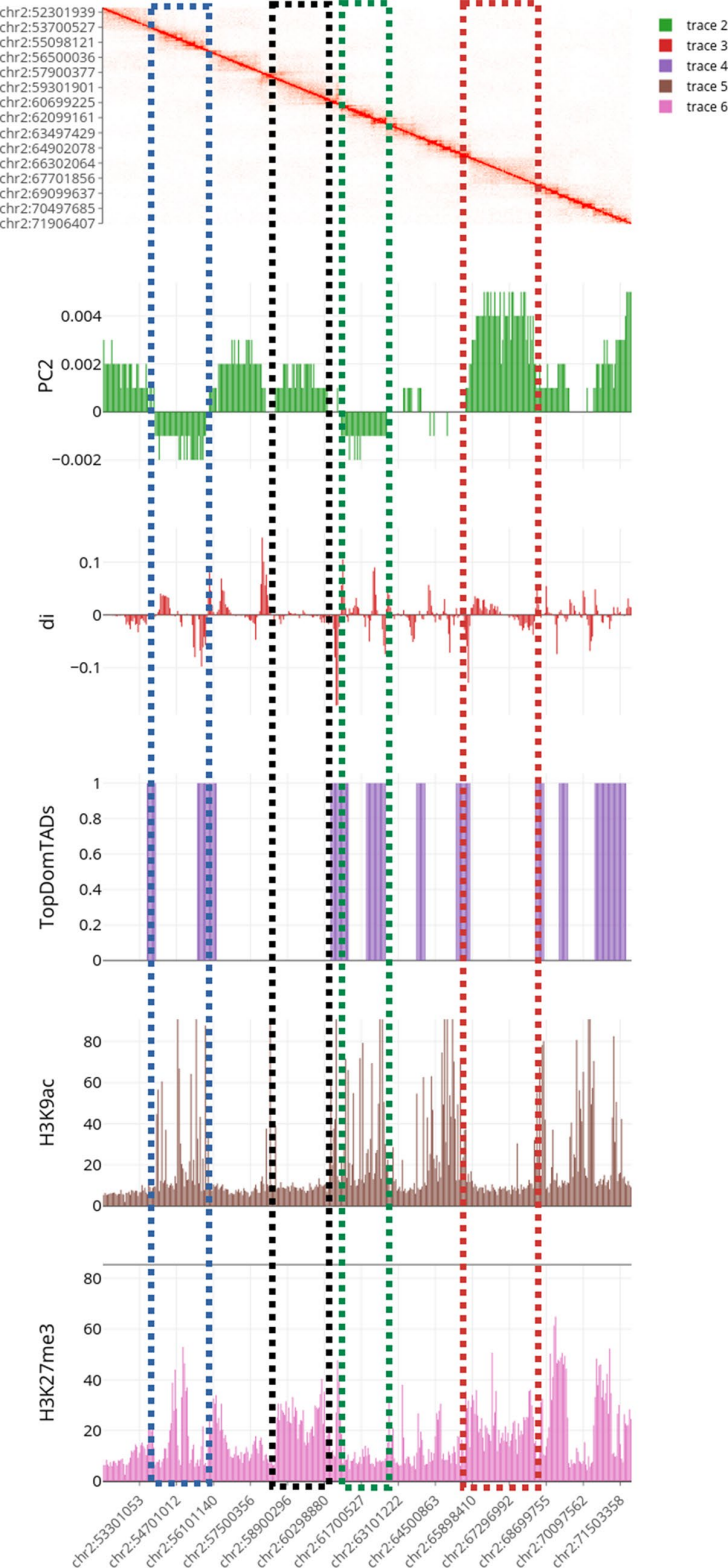
Multilayer visualization of the region 51902204–71950291 of chromosome 2 (replicate GSM1608509). From the top, the first track shows the normalized contact matrix as a heatmap, where the color intensity is proportional to the strength or the interaction. The green track shows the eigenvector of the second principal components (PC2) that define the putative A/B compartments. The red track displays the directional indexes *di* (that helps in defining TADs). The purple track shows the TADs boundaries as detected by TopDomTADs. The two remaining tracks show the RPM normalized coverage for H3K9Ac (in brown) and H3K4me2 (in pink) histone marks. The H3K9Ac and H3K4me2 enriched regions exhibit a profile similar to PC2 track, indicating that it overall correlates with the 3D organization of the chromosomes in these regions.

**Figure 7:**
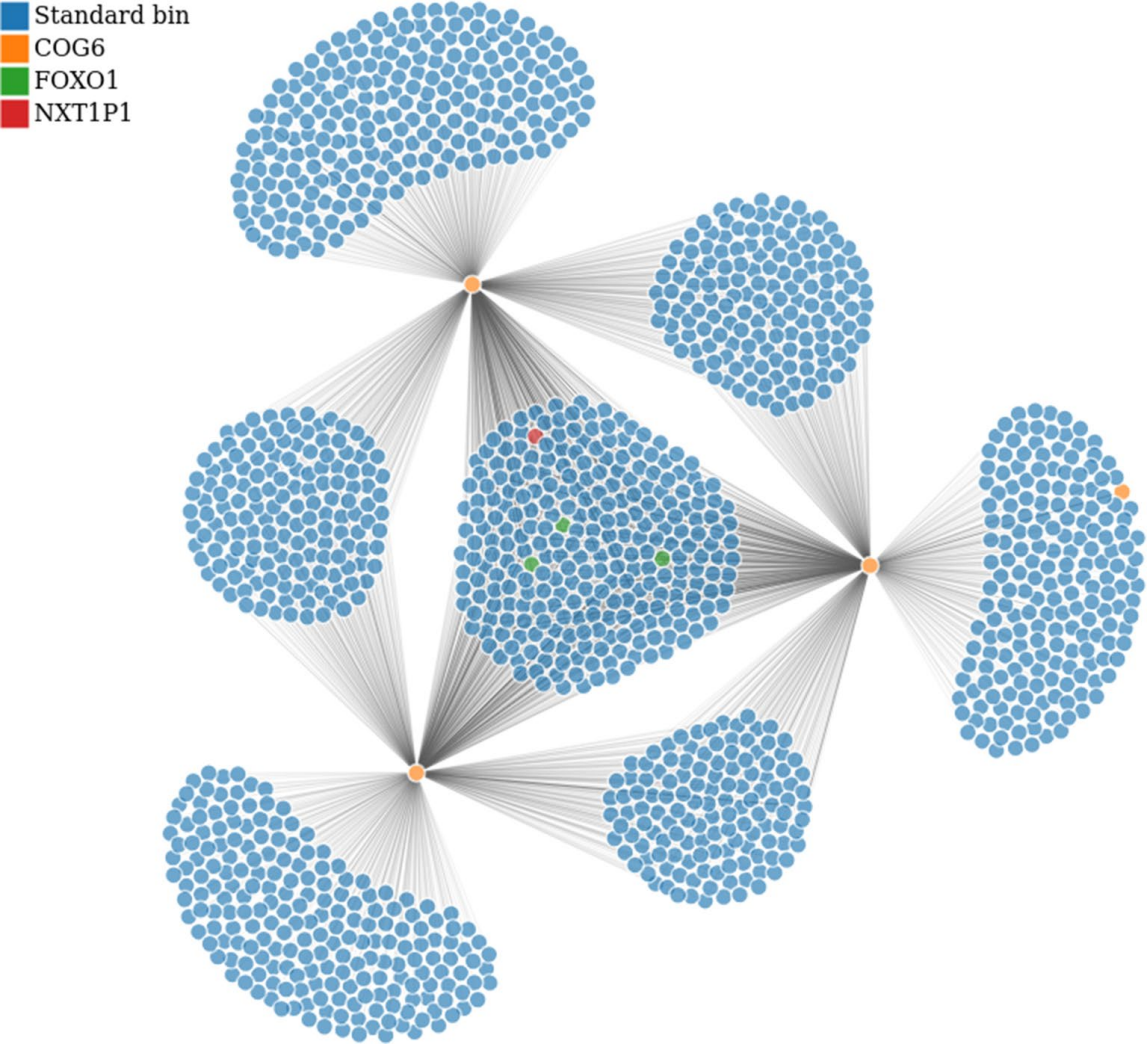
Bin to bin interaction network (evaluated with low stringency). The interaction network was built starting from the region in Tab. 1 containing COG6 gene (bins in orange) and retrieving all interactions within chromosome 13. Additionally, we highlighted the bins containing FOXO1 and NXT1P1, in green and red respectively. This analysis has been performed on GSM160850 replicate.

We illustrate the capability of HiCeekR in analyzing Hi-C data using a dataset from the lymphoblastoid cell line (GM12878) produced from the blood of a female donor, freely available (in FASTQ format) from Gene Expression Omnibus (GEO) (accession number GSE62742). The dataset contains seven biological replicates (including GSM160850 replicates used in the illustrative **Figures 2**, **6**, and **7**), each of them obtained from approximately 25 millions of cells prepared with standard Hi-C library protocol digested with HindIII. The runs were sequenced using Illumina HiSeq 2000 to produce 2 × 75 paired-end sequences for each library, see Grubert et al. (2015) for details.

Before starting the analysis with HiCeekR, the sequence files were independently aligned to the human reference genome using HiCUP and the hicupmapper script.

In particular, low quality reads (i.e. reads with more than one mismatch in the first 28 bases or the ones with a summed Phred quality score lesser than 70 for all mismatched positions) were removed and only uniquely mapped reads were reported in the BAM files. Duplicated reads were marked using the Picard tools with MarkDuplicates (version 2.18.4). Such BAM files constitute the starting point of the HiCeekR analysis.

We also downloaded a series of ChIP-Seq and RNA-Seq datasets on the same cell line from the ENCODE portal, to illustrate the capability of HiCeekR in integrating other omic data. In particular, we selected already aligned BAM files for the following histone modifications: H3K9Ac, H3K9me3, H4K20me1, H3K27me3, H3K36me3, H3K4me2, H3K4me3, H3K79me2 (ENCSR447YYN series from Bradley Bernstein laboratory at Broad Institute). For simplicity, using the samtools (version 1.9), we merged the three replicates of each modification into a single BAM file, that was sorted and indexed. From RNA-Seq experiment (ENCFF383EXA series from California Institute of Technology or GEO accession number GSE33480) we downloaded the normalized gene expression values and obtained a single two-column tab-delimited file with the gene identifier in the first column and fragments per kilobase of transcript per Million mapped reads (FPKM) in the second one.

All the analyses were performed using as reference genome GRCh37.p13 (https://www.ncbi.nlm.nih.gov/assembly/GCF_000001405.25/) and the gene annotation file obtained from GENCODE gencode.v19.annotation (ENCSR884DHJ).

### HiCeekrR Computational Analysis

After creating the new project, we independently analyzed the seven replicates by selecting the corresponding BAM file from the Pre-processing module. For each sample, we selected the reference genome, the cutting enzyme in the *cut site* text-box (*HindIII* site “AAGCTT”), and an *overhang* parameter of 4 bp. Then, we executed the pre-processing and we set 50,000 bp as bin resolution for the rest of the analysis. Therefore, for each BAM file, HiCeekR created a specific folder inside the project folder where the results were saved.

The fragment length and the reads-orientation plots (see **Figure 2**—before filtering) were used to explore the presence of artifacts. We noticed that all the seven replicates show a self-circle spike close to 2^8^  =  256 bp. By using the *Filtering* module, for each BAM file, and setting *min.inward* parameter equal to 1,000 bp, we filtered-out the spike because we are not interested in reads falling in the same restriction fragment. At the same time, since we did not notice dangling-end artifacts, we did not set any *min.outward* threshold to remove it. **Figure 2**—after filtering—illustrates the effect of the applied filtering. Note that, within HiCeekR the figure is interactive, a slide bar allows the user to choose the cut-off directly on the plot.

Afterward, we executed the Binning module using default settings. HiCeekR automatically loaded all required files from the sample under analysis and processed for all the chromosomes. At the end of this step, the detected interactions are shown in the results panel (and saved in the corresponding folder), as bin-to-bin interaction tables.

For this illustrative example, we decided to investigate only chromosomes: 1, 2, 3, 13, 14, 16, since they were previously studied in Martin et al. (2015). Therefore, we selected the corresponding target chromosomes inside “*chromosome of interest*” *selection box* and ticked the *selective bin table* check-box inside the *Export* panel to continue the analysis.

From the Normalization module, we selected the ICE normalization method and set the *Window.high* parameter equal to 0.02 in combination with the *ignore.low = 0* parameter to ignore the low abundance bins. Moreover, to avoid the *NA* values produced by the DiffHiC implementation, we also selected the “Set NA to min” check-box. In such a way HiCeekR sets all the *NAs* to the *min* of the matrix. Afterward, we exported the normalized contact matrix for the chromosomes of our interest. Note that these normalized matrices constitute the starting point of the post-processing analysis.

For brevity, here we illustrate only two cases of usage for the Post-processing: *i)* We first identified compartments and TADs, then we integrated them with ChIP-Seq data, and visualized a region of interest (as in **Figure 6**), *ii)* We converted the normalized contact matrix in a network of interactions for some regions of interests (as in **Figure 7**), then we identified the genes located in each interacting bin and performed gene functional analysis. In this latter case, we also added the gene expression values from RNA-Seq data.

For the first case, we used the PCA module on the normalized contact matrix. Afterward, we used the directionality index module to determine the directional index *di* and TopDomTADs with *Window Size = 20* that provides us a list of TADs boundaries in BED format. Then, we used the EpigeneticFeature module to process ChIP-Seq dataset and compute the normalized coverage at the same genomic resolution of the HiC-Seq analysis (i.e. over bins). Using the *select bin Table file* selector, we chose the BED file corresponding to the chosen bin resolution and the chromosome of interest (here we chose chromosome 2). Then, in the first sub-panel, we selected the first BAM file for the ChIP-Seq data, e.g. the *H3K9Ac* BAM file, through the *BAM file path* selector, and we associated “H3K9Ac” as track label. By checking the *add* checkbox, we added a second track without replacing the previous one. We repeated this operation for H3K9me3, H4K20me1, H3K27me3, H3K36me3, H3K4me2, H3K4me3, H3K79me2. At the end of the process for each sample, HiCeekR generated a vector containing the raw coverage (number of mapped reads) in the bins. Using the second sub-panel, we exported the coverage for all samples as a combined table. To do this, we chose the file name through the *file name* text input and the normalization strategy to use (in the *normalization* checkbox). For this case study, we performed the *RPM* normalization and saved the results using the *export table* button.

Using heatmap module (*layout*), we selected the normalized contact matrix by the *contact matrix* input file widget and we focused the attention on the region 51902204–71950291 of chromosome 2, as illustrative example. From the same panel, we added four additional tracks. In particular, we selected in the first slot the PCA file obtained from the pca module. Since this file contains multiple columns (corresponding to the eigenvectors of the principal components), we selected the eigenvector corresponding to the second principal component (PC2). Note, PC1 or PC2 are usually used to describe compartments, the specific choice depending on the size of the region of interested and the resolution of the analysis. In the second slot, we loaded the directionality index *d* *_i_* file. After that, we added the bed track of the TADs boundaries as produced by the TopDomTADs. Then, we added the two epigenetic tracks (produced in EpigeneticFeatures module) selecting “H3K9Ac” and “H3K27me3” features columns as an illustrative example. At the end of these uploads, we are able to visualize all the tracks by flagging the *active* checkbox in each slot panel (see **Figure 6**).

In the second case, we used the network module in the Visualization panel and focused the attention on the regions investigated in Martin et al. (2015), listed in **Table 1**. Note that since the regions in **Table 1** are often larger than the bin size chosen for this analysis, each region can correspond to a few bins.

**Table 1:** The list of regions identified in Martin et al. (2015) (as chromosome, start, end of the region, and the most relevant genes therein located).

Chr	Start	End	Genes
Chr1	197,473,879	197,744,623	*DENND1B*
Chr3	27,757,440	27,764,206	*EOMES*
Chr13	40,229,764	40,326,765	*COG6*
Chr14	69,262,513	69,454,180	*ZFP36L1*, *ACTN1*
Chr16	11,022,748	11,036,257	*DEXI*

To this purpose, we first selected the normalized contact table (using the *contact table* input file widget), then the gene annotation file (using *Annotation* file input), finally we added the RNA-Seq gene expression data, by selecting the specific file in the *Expression data* file input. By pressing the *set input* button HiCeekR loaded the data and moved into the second tab panel (*show*). Inside this tab panel, we selected the chromosomal coordinates given in **Table 1** (analyzing them individually). For all the interested regions, we set the *normValue* to 0.01 and checked the *global* checkbox (in the left panel). Since the focus of the study was to enlighten long-range interactions, we excluded from the visualization all those regions with a bin distance lower than eight bins, by checking the *intra Chr* checkbox and setting the *min bin distance* text box to 8. Then, HiCeekR visualized the network (see **Figure 7**) and produced three interactive panel-tables (i.e. *Interactions*, *Genes*, and *Functional*), as mentioned in *Pre-Processing*. Within panel-tables *Interactions*, we ranked all the interactions by the interaction strength from the strongest (higher contact matrix value) to the weakest (lower contact matrix value). Therefore, we identified the strongest bin to bin interactions together with the genes therein contained. For the functional analysis, we selected the *hsapiens* database in the *organism* select box.

### Analysis Results

Results of the first analysis are summarized in **Figure 6**, where the short p-arm of chromosome 2 (chr2:51,000,000–71,000,000) is displayed in a multi-layer view. The figure includes the normalized contact matrix (on the top) and, in order, the PC2 eigenvector (as a green track), the *di* indices (as a red track), the TADs boundaries as detected by TopDom (as a purple track), and the RPM normalized tracks of the histone marks H3K9Ac, H3K27me3 (as brown and pink tracks), which are associated to transcribed an repressed chromatin, respectively. We highlighted a correlation between the typical rectangular block-shapes in the heatmap and the PC2 loadings allowing detecting the A/B compartments (territories) and categorizing also the TADs thanks to the directionality indexes *di*. Additionally, the histone mark tracks allow us to better characterize the chromatin structure within each pattern. A clear correlation between distinct A/B compartments and the H3K9Ac and H3K27me3 enriched regions is shown at the selected chromosomal region (**Figure 6**).

For the second analysis, we report the independent analysis of the regions in **Table 1**. First of all, we noticed that the regions identified in Martin et al. (2015) are often among the strongest interactions (top positions after ranking by strength) identified in our analysis.

In particular, from the panel-table *Interactions*, we easily identified the following gene-bins interactions, where gene-bins means the bins overlapping or containing a given gene. Recall that, based on the chosen resolution and the length of the gene body, each bin might contain few genes, or a given gene might be associated with few bins. We identified that the *EOMES-bins* has multiple strong interactions within chromosome 3, as previously reported Martin et al. (2015). Out of them, the *EOMES-bins* was found to interact with the *AZI2-bins* (such interaction was confirmed for all replicates with strength spanning from 0.020 to 0.025 in the normalized matrices). Additionally, we confirmed the interaction between the *COG6-bins* and the *FOXO1-bins* within chromosome 13, although it is weak (about 0.01 in the normalized matrices). By contrast, we found that the *COG6-bins* presents a strong interaction with the *NXT1P1-bins* (chr13:39697243-39750825) (about 0.017 in the normalized matrices). Such case is illustrated in **Figure 7**, where the *COG6-bins* are depicted in yellow, and the *NXT1P1-bins* and *FOXO1-bins* are depicted in red and green, respectively. Moreover, the *DEXI-bins* on chromosome 16 shows a strong interaction with the *RMI2-bins*, as reported in Martin et al. (2015). Indeed, this interaction was found with strength from 0.0344 to 0.033 in the normalized matrices, being among the strongest interactions that this region shows with distant regions. This region seems also to interact with the *ZC3H7A-bins*, although this interaction is weaker (value close to 0.01) than others. On the other hand, when moving to chromosome 1, the *DENND1B-bins* shows a strong interaction with the *LHX9-bins* (with normalization matrix values spanning from 0.020 to 0.030). Finally, on chromosome 14, we partially confirmed the interaction between the bins containing the *ZFP36L1* and *ACTN1* genes and the *ZFYE26-bins*. This interaction was observed only in a subset of replicates, and, when detected, it shows low strength (normalized value of about 0.01).

From the panel-table *Genes*, we found that, according to the RNA-Seq data, all above mentioned interacting genes are expressed except *LHX9* and show variable expression levels in RNA-Seq: *ZC3H74* gene has the highest RPKM value (186.55), *ZFP36L1*, *ZFYVE26*, and *AZI2* genes show high expression (59.03, 48.67, 37.93 respectively), while *DENDD1B*, *EOMES*, *FOXO1*, *ACTN*, *DEXI*, and *RMI2* genes show a lower level of expression (ranging from 4.21 to 6.87).

Finally, the most interesting results of the functional enrichment analysis performed on the genes interacting with regions in **Table 1** are given in **Table 2**. We can see that *DENND1B* gene, which codifies for a guanine nucleotide exchange factor (GEF) acting as a regulator of T-cell receptor (TCR) internalization in T-cells interacts with LHX9, ATP6V1G3, C1ORF53 genes. They show significant enrichment of binding sites for the transcription factor T-bet, that is a master regulator of the T-helper 1 (Th1) cell development (Kallies and Good-Jacobson, 2017). The zinc-finger *ZFP36L1* gene interacts with *RAD51B* and *ACTN1* genes, which codify for proteins involved in homologous recombination and cell migration, respectively (Lio et al., 2003; Yamaji et al., 2004). Remarkably, the *AZI2* gene, which interacts with the *EOMES* gene, is an important activator of *NF-kB* signaling as also reported in Martin et al. (2015). It shows binding sites for the *FOXJ2* transcription factor, which strictly correlated with *NF-kB* signaling (Lin et al., 2004).

**Table 2:** Results obtained from the functional analysis; the table contains significant terms identified starting from the list of genes contained in the bins strongly interacting with the regions examined by network construction.

Region	term.id	dm	term.name	intersection	*p*-value
DENND1B	TF:M08355	tf	Factor: HOXB2:T-bet	LHX9, ATP6V1G3, C1ORF53	0.0195
EOMES	TF:M08290_1	tf	Factor: FOXJ2:Elf-1	AZI2, ZCWPW2	0.0053
EOMES	TF:M03979_1	tf	Factor: ETV1	AZI2, ZCWPW2	0.0306
EOMES	TF:M07287_1	tf	Factor: FOXO3A	AZI2, ZCWPW2	0.0362
ZFP36L1,ACTN1	CORUM:260	cor	RAD51B-RAD51C complex	RAD51B	0.0497
ZFP36L1,ACTN1	CORUM:4025	cor	Affixin-actinin (alpha) complex	ACTN1	0.0497

### Computational Costs

The analysis of this case-study was executed on an Intel i7-7700HQ processor, with 32Gb RAM system (64bit architecture) on Ubuntu 18.04 LTS, with R version 3.6.1 and Shiny 1.3.2. Other relevant packages are listed in the github page.

The most computationally expensive step is the pre-processing of Hi-C data which requires approximately 20 to 25 min for processing a single BAM file of approximately 150 million of reads. For the binning step, performed on large chromosomes such as human chromosome 1 or 2, with bin size 50,000 bp, the elapsing time is about 3 to 5 min including the output file storage. While for the normalization step the required time is about 30 s. The identification of TADs requires 2 to 5 min per chromosome, depending on the methods and the size of the chosen chromosome. Another time demanding step is the import of indexed ChIP-Seq BAM files that can even take a couple of hours for samples with very high depth such as those obtained after merging different replicates. The computational time is clearly reduced when working with a specific chromosome or at lower bin resolutions or with organisms with smaller genomes.

### Software Availability and System Requirements

HiCeekR is freely available as source code package on GitHub (https://github.com/lucidif/HiCeekR), where future releases will be also posted. Moreover, issues and problems can be submitted to the HiCeekR developers through the github issues page to contribute to the development of future releases. The github page also includes a detailed user manual where all HiCeekR modules are described and the data used in the current study that can be used as training example. The current version of HiCeekR was developed and tested on Ubuntu 16/18 and macOS 10.13, using R environment version 3.6.1, and the latest releases of R packages is reported on the github page as Session Info. System requirements strongly depend on the size of the reference genome, sequencing depth and, in particular, on the bin resolution. However, minimal system requirements are Intel i5 4th generation processor and 16Gb RAM.

## Conclusions

Despite the relevance of Hi-C data and the availability of several packages for performing specific steps in their analysis, only a few comprehensive and user-friendly tools have been developed during the last years (Schmid et al., 2015; Caudai et al., 2018; Wolff et al., 2018). Thanks to its GUI, HiCeekR provides an easy-to-use way to analyze this data type, specifically designed to guide researchers lacking specific training in scientific programming through the different computational steps. Moreover, it also provides multiple approaches for integrating Hi-C data with other omic datasets and a wide series of interactive graphical outputs that can significantly support researches in the interpretation of the huge amount of data produced during Hi-C experiments. The major capabilities of HiCeekR are illustrated by analyzing a publicly available dataset, and integrating ChIP-Seq and RNA-Seq dataset.

Moreover, HiCeekR is implemented in a modular structure. Therefore, other approaches available in literature could be easily encapsulated in further releases. In this regard, an interesting extension is the one proposed by Merelli et al. (2015). In this latter case, by using NuChart tool they build multiple gene-centric graphs starting from Hi-C and transcription data, allowing additional statistical investigations, thanks to the graph-based approach. Such an approach can complement HiCeekR network approach to provide a wider range of methods. It is also clear that post-processing analysis constitutes one of the aspects where artificial intelligence approaches can still greatly contribute to the elucidation of chromatin structure and gene regulation interplay, therefore several other algorithms are expected to be available soon. Hence, we expect that HiCeekR will growth by expanding the number of methods available.

On the other hand, although HiCeekR already implements several methods to facilitate Hi-C data analysis, much work still needs to be done to speed-up the time-demanding computations required for carrying out some specific steps, such as the pre-processing and binning. A possible improvement is the implementation of a parallel version of the algorithms used in HiCeekR or the split-up of the computations on multiple cores/CPUs. In this regards, a good example is given by the NuChart-II R packages, where particular attention is reserved for the implementation of parallel routines for Hi-C data analysis (Merelli et al., 2013; Tordini et al., 2017).

Last but not least, HiCeekR can be improved to better supporting computational reproducible research. Indeed, thanks to its GUI approach, HiCeekR guides the user to perform a complete analysis of Hi-C data, automatically storing input/output data. Despite this is very helpful from the user point of view, it does not provide reproducible research functionalities yet. As mentioned in (Russo et al., 2016b), it is known that the problem of computational reproducibility is very challenging for tools based on GUI, since it becomes hard to precisely trace all the steps/parameters of the analysis workflow when the user can apply a point-and-click approach. However, in the same spirit such that (Russo et al., 2016a) was extending RNASeqGUI (Russo and Angelini, 2014) in the direction of reproducible research, we plan to implement multiple functionalities to automatically produce a comprehensive analysis report incorporating all the executed code and the results (as tables and figures).

## Data Availability Statement

Publicly available datasets were analyzed in this study. This data can be found here: https://www.ncbi.nlm.nih.gov/geo/query/acc.cgi?acc=GSE62742. Accession number: GSE62742.

## Author Contributions

LF designed and implemented HiCeekR, performed analysis of the real cases, and drafted the manuscript. DR contributed to the design and implementation of HiCeekR and wrote the manuscript. MG and MM contributed to the discussion of the real data analysis. CA contributed to the design of HiCeekR. MM and CA guided and supervised all phases of HiCeekR development and wrote the manuscript. All authors read and approved the manuscript.

## Funding

This work was partially supported by the Italian Flagship project (Epigen) and the Regione Campania Project ADViSE.

## Conflict of Interest

The authors declare that the research was conducted in the absence of any commercial or financial relationships that could be construed as a potential conflict of interest.

The reviewer PL declared a past co-authorship with one of the authors CA to the handling editor.
